# The Glycosylation Site of Myelin Oligodendrocyte Glycoprotein Affects Autoantibody Recognition in a Large Proportion of Patients

**DOI:** 10.3389/fimmu.2019.01189

**Published:** 2019-06-11

**Authors:** Iris Marti Fernandez, Caterina Macrini, Markus Krumbholz, Paul J. Hensbergen, Agnes L. Hipgrave Ederveen, Stephan Winklmeier, Atay Vural, Asli Kurne, Dieter Jenne, Frits Kamp, Lisa Ann Gerdes, Reinhard Hohlfeld, Manfred Wuhrer, Tania Kümpfel, Edgar Meinl

**Affiliations:** ^1^Biomedical Center and University Hospitals, Institute of Clinical Neuroimmunology, Ludwig-Maximilians-Universität München, Munich, Germany; ^2^Department of Neurology and Stroke, Hertie Institute for Clinical Brain Research, University of Tübingen, Tübingen, Germany; ^3^Center for Proteomics and Metabolomics, Leiden University Medical Center, Leiden, Netherlands; ^4^Koç University School of Medicine, Istanbul, Turkey; ^5^Department of Neurology, Hacettepe University, Ankara, Turkey; ^6^Comprehensive Pneumology Center (CPC), Institute of Lung Biology and Disease, Helmholtz Zentrum München, Munich, and Max Planck Institute of Neurobiology, Planegg, Germany; ^7^Biomedical Center (BMC), Metabolic Biochemistry, LMU Munich, Munich, Germany; ^8^Munich Cluster for Systems Neurology (SyNergy), Munich, Germany

**Keywords:** myelin oligodendrocyte glycoprotein (MOG), glycosylation, autoantibody recognition, mass-spectrometry, demyelination

## Abstract

Autoantibodies to myelin oligodendrocytes glycoprotein (MOG) are found in a fraction of patients with inflammatory demyelination and are detected with MOG-transfected cells. While the prototype anti-MOG mAb 8-18C5 and polyclonal anti-MOG responses from different mouse strains largely recognize the FG loop of MOG, the human anti-MOG response is more heterogeneous and human MOG-Abs recognizing different epitopes were found to be pathogenic. The aim of this study was to get further insight into details of antigen-recognition by human MOG-Abs focusing on the impact of glycosylation. MOG has one known N-glycosylation site at N31 located in the BC loop linking two beta-sheets. We compared the reactivity to wild type MOG with that toward two different mutants in which the neutral asparagine of N31 was mutated to negatively charged aspartate or to the neutral alanine. We found that around 60% of all patients (16/27) showed an altered reactivity to one or both of the mutations. We noted seven different patterns of recognition of the two glycosylation-deficient mutants by different patients. The introduced negative charge at N31 enhanced recognition in some, but reduced recognition in other patients. In 7/27 patients the neutral glycosylation-deficient mutant was recognized stronger. The folding of the extracellular domain of MOG with the formation of beta-sheets did not depend on its glycosylation as seen by circular dichroism. We determined the glycan structure of MOG produced in HEK cells by mass spectrometry. The most abundant glycoforms of MOG expressed in HEK cells are diantennary, contain a core fucose, an antennary fucose, and are decorated with α2,6 linked Neu5Ac, while details of the glycoforms of MOG in myelin remain to be identified. Together, we (1) increase the knowledge about heterogeneity of human autoantibodies to MOG, (2) show that the BC loop affects recognition in about 60% of the patients, (3) report that all patients recognized the unglycosylated protein backbone, while (4) in about 20% of the patients the attached sugar reduces autoantibody binding presumably via steric hindrance. Thus, a neutral glycosylation-deficient mutant of MOG might enhance the sensitivity to identify MOG-Abs.

## Introduction

Autoantibodies against myelin oligodendrocyte glycoprotein (MOG) detected in cell-based assays occur in a proportion of patients with inflammatory CNS diseases. High levels of such autoantibodies were initially detected in pediatric patients (1–3), then also in adults, and MOG-Abs are implicated in prognosis and therapy optimization (4–11). MOG-Abs are assumed to be pathogenic based on *in vitro* experiments (12–15) and injection of total IgG from anti-MOG positive patients into experimental animals (16–19). We have recently reported that affinity-purified MOG-Abs from two patients who show cross-reactivity to rodent MOG were pathogenic upon transfer into EAE animals by two different mechanisms, namely by enhancing T cell activation of cognate T cells and by inducing MS type II like demyelination when the blood-brain barrier is breached (20).

MOG is exposed on the outside of intermodal myelin; the crystal structure of the extracellular part of mouse (21) and rat MOG (22) allowed the modeling of human MOG (23). The antigen-binding fragment (Fab) of the prototype anti-MOG mAb 8-18C5 was crystallized together with the extracellular part of MOG and this revealed that the FG loop (aa101-108) of MOG, which constitutes an IgV-like fold, makes the dominant contribution to binding of this particular mAb (22). A subsequent study showed that the amino acids His103 and Ser104 are essential for binding of the mAb 8-18C5 and also for the polyclonal anti-MOG IgG induced upon MOG DNA-vaccination of BALB/c and SJL/J mice (24). In contrast to these rodent models, the anti-MOG Abs in human patients are more heterogeneous and most of the patients recognize epitopes that are different from that of the prototype mAb 8-18C5 (23). Also, the epitopes of MOG-Abs affinity-purified from two patients were found to be pathogenic upon transfer into rats and they differed in their fine-specificity from the mAb 8-18C5 (20).

The aim of this study was to get further insight into details of antigen recognition of human autoantibodies against MOG. Specifically, we analyzed here the impact of the glycosylation site of MOG on antibody binding. In principal, glycosylation of an antigen can have different, even opposing effects on antibody binding. For example, recognition of contactin by autoantibodies from 3/4 patients with chronic inflammatory demyelinating polyneuropathy depended on specific contactin *N*-glycosylation (25). In contrast, glycosylation of the Env-protein of the immunodeficiency viruses HIV and SIV at multiple sites blocks antibody binding and is an immune evasion strategy of these viruses in infected individuals (26, 27). Now broadly neutralizing Abs to HIV are a therapeutic perspective, but such Abs have to accommodate and avoid glycans, while some of them recognize glycan-dependent epitopes (28).

MOG has one N-linked glycosylation site, N31 (23). It was previously observed that when this asparagine was mutated to aspartate (N31D), the MOG-recognition of some patients was altered (1, 23, 29). It was unclear, however, whether this altered binding is due to the introduction of the negatively charged aspartate or due to the abrogation of glycosylation. We addressed this issue here by generating a neutral glycosylation deficient mutant of MOG (N31A) and comparatively analyzed the anti-MOG reactivity in a total of 27 anti-MOG positive patients to wild type MOG and the different glycosylation-deficient variants of MOG. Thereby we found that the different mutations of the glycosylation site affect the antigen recognition in 15/27 patients and noted seven different patterns of antigen-recognition of variants of the glycosylation site. We applied mass spectrometry to determine the glycoforms of MOG in HEK cells, because HEK cells are the preferred expression system to analyse MOG-Abs in cell based-assays (12, 30–33). Our data extend our knowledge about the heterogeneity of human autoantibodies to MOG, indicate that the glycosylation site affects antigen-binding in a large proportion of patients and that the glycan attached to MOG is a steric hindrance for antigen recognition in some patients.

## Materials and Methods

### Patients

This study included sera of 27 adult patients with different inflammatory CNS diseases and antibodies to cell-bound MOG (Table 1). We give the original diagnosis in Table 1. It is currently discussed whether patients with MOG-Abs constitute a separate disease entity. Some of our patients had got the initial diagnosis of MS, but typical MS patients do not have MOG-Abs (30). Nevertheless, in many studies patients have been described who met the diagnostic criteria of MS and were MOG-Ab positive (4, 7, 10). These may be atypical cases or patients fulfilling the criteria of MS, but with a specific phenotype (34), mostly with a low intensity of anti-MOG reactivity. Informed consent was obtained from each donor according to the Declaration of Helsinki and the ethical committee of the medical faculty of the LMU approved this study.

**Table 1:** Details of the anti-MOG positive patients.

**Patient ID^**a**^**	**Diagnosis^**b**^**	**Treatment at the point of blood drawn**
1	LETM	None
2	MS	Teriflunomide
3	MS/NMOSD	Steroids + Teriflunomide
4	ADEM	None
5	CIS	None
6	Relapsing ON	None
7	MS	Natalizumab
8	NMOSD	Cyclophosphamide
9	ON	None
10	RON	Rituximab
11	RON	Rituximab
12	ON	Azathioprine
13	NMOSD	Azathioprine
14	BON	Azathioprine
15	Relapsing encephalomyelitis	Steroids + Plasmapheresis
16	Relapsing ON	None
17	Relapsing encephalomyelitis	Azathioprine
18	Relapsing encephalomyelitis	Steroids
19	MS	Glatiramer acetate
20	NMOSD	Azathioprine
21	NMOSD	None
22	Relapsing ON	Azathioprine
23	NMOSD	Glatiramer acetate
24	Monophasic encephalitis	None
25	Relapsing ON	None
26	Relapsing ON	None
27	NMOSD	Steroids

a Some patients have been previously described in more detail: Patient 17 in Spadaro et al. (29); patients 7 and 19 in Spadaro et al. (34), and patients 15, 16, 22, 23, 25, 26, and 27 in Spadaro et al. (20).

b *We give the original diagnosis; It is currently discussed whether patients with MOG-Abs constitute a separate disease entity (8, 11, 35, 36). LETM, longitudinal extensive transverse myelitis; NMOSD, neuromyelitis optica spectrum disorder; ADEM, acute disseminated encephalomyelitis; CIS, clinically isolated syndrome; ON, optic neuritis; RON, recurrent optic neuritis; BON, bilateral optic neuritis*.

### Molecular Cloning and Transfection

Full-length human MOG was subcloned into the pEGFP-N1 plasmid (CLONTECH Laboratories, Mountain View, CA, USA). This construct comprises a C-terminal enhanced GFP (EGFP)-tag. Using the QuickChange Site-Directed Mutagenesis Kit (Stratagene, Santa Clara, CA, USA), point mutations were induced into MOG. The oligonucleotides used were: 5′-CAT ATC TCC TGG GAA GGA CGC TAC AGG CAT GGA GG-3′ (N31D) (23),5′-CAT ATC TCC TGG GAA GGC AGC TAC AGG CAT GGA GG-3′ (N31A), and the corresponding reverse complementary oligonucleotides. The sequences of the purified plasmids were confirmed. HeLa cells were transfected transiently using jetPRIME (Polyplus, Illkirch, France) according to the instruction of the manufacturer, expressing MOG, N31D, or N31A fused C-terminally to EGFP. Surface expression of each of the MOG-constructs was confirmed by FACS-staining using a recombinant version of the anti-MOG mAbs 8-18C5 with a human IgG1 as Fc part (20, 37), which we call r8-18C5.

### Determination of Reactivity to MOG Variants in a Cell-Based Assay

For detection of serum antibodies, HeLa cells transiently transfected with hMOG and its variants were suspended in FACS buffer (1% FCS in PBS). The cells were incubated with a 1:50 serum dilution or mAb r8-18C5 (0.5 μg/ml) for 45 min at 4°C and washed three times in FACS buffer. The cells were then incubated with a 1:500 dilution of a biotin-SP conjugated goat anti-human IgG (Jackson ImmunoResearch, West Grove, PA, USA) for 30 min at 4°C, washed three times, and incubated with Alexa Fluor 647-conjugated streptavidin (Jackson ImmunoResearch) at a dilution of 1:2000. Finally, the cells were washed three times and suspended in a 1:2000 dilution of propidium iodide in PBS. Dead cells were excluded by positive propidium iodide staining. For the determination of anti-MOG reactivity, we gated on cells with a fluorescein isothiocyanate fluorescence (FITC) intensity above 500 and determined their mean channel fluorescence intensity (MFI) in the allophycocyanin channel (APC). Cells transfected with the mutants, wild type MOG, and with EGFP only were always measured together in the same experiment. To quantify the reactivity to the MOG variants, MFI ratio was calculated as (MFI to the MOG variant-EGFP)/(MFI to EGFP). This MFI ratio reflects properties of the autoantibodies, both amount and affinity.

### Deglycosylation

HeLa cells transfected with MOG-EGFP constructs were lysed at 4°C for 1 h in RIPA buffer (150 mM NaCl, 1% NP-40, 0.5% sodium deoxycholate, 50 mM Tris pH8, 0.1% SDS) containing complete protease inhibitor mixture (Roche Applied Science, Penzberg, Germany). The lysate was then pelleted, and the supernatant was analyzed. For deglycosylation, the supernatant was digested with PNGaseF (New England Biolabs, Ipswich, MA) in Glycoprotein Denaturing Buffer (New England Biolabs), Glycobuffer 2 (New England Biolabs) and 1% NP40 (New England Biolabs) at 37°C overnight; Proteins (digested or undigested) were analyzed by SDS-PAGE. The proteins were electroblotted onto a PVDF membrane and detected by Western blot with an anti-GFP-HRP conjugated antibody (Genetex, Irvine, CA, USA) and developed using the Immobilon Western kit used (Millipore, Burlington, MA, USA) and the Odyssey Fc Imaging system (LI-COR, Bad Homburg, Germany).

### Production of Recombinant MOG

We produced a recombinant version of the extracellular domain (ECD) of human MOG (20) in HEK293-EBNA cells and added at the C-terminus instead of the first transmembrane region a HisTag and an AviTag using the pTT5 vector (38). HEK293-EBNA cells were transfected, cultured under serum-free conditions with the FreeStyle293 Expression Medium (Thermo Fisher Scientific, Waltham, MA, USA). The secreted ECD of MOG was purified with a His Trap HP column (GE Healthcare, Uppsala, Sweden). With this expression system we produced the ECD of the wild type MOG and a glycosylation-deficient variant (T33N). Folding of the purified proteins (0.2 mg/ml) was analyzed by circular dichroism using a Jasco J-810 Spectropolarimeter (JASCO Corporation, Tokyo, Japan). Data were corrected for the spectrum of the buffer alone.

### Preparation of Ethyl Esterified Released *N*-Glycans From Recombinant MOG

An SDS-PAGE gel band corresponding to HEK cell derived MOG (15–20 μg, migrating at ~21 kDa) was reduced, alkylated and subsequently treated with *N*-glycosidase F (PNGase F; Roche Diagnostics, Mannheim, Germany) to release the *N*-glycans, as described previously (39). Additionally, 5 μg of HEK derived MOG was denatured and incubated overnight with PNGaseF in-solution at 37°C (40, 41). Released *N*-glycans were subjected to the selective ethyl esterification of sialic acids, thereby introducing mass differences of +28.03 Da and −18.01 Da for α2,6-linked *N*-acetylneuraminic and α2,3 *N*-acetylneuraminic acid, respectively (40). Briefly, released glycans were incubated with the derivatization reagent (250 mM 1-ethyl-3-(3-(dimethylamino)propyl)carbodiimide and 250 mM 1-hydroxybenzotriazole in ethanol) and incubated for 60 min at 37°C. The derivatized glycans were enriched by cotton hydrophilic-interaction liquid chromatography (HILIC)–solid-phase extraction (SPE) as described before (42) and eluted in water.

### MALDI-TOF(/TOF)-MS(/MS) Analysis of Released Glycans

MALDI-TOF-MS analysis was performed on an UltrafleXtreme (Bruker Daltonics) operated under flexControl 3.3 (Build 108; Bruker Daltonics). Two and 5 μL of the enriched ethyl esterified glycans were spotted on a MALDI target (MTP AnchorChip 800/384 TF; Bruker Daltonics) together with 1 μL of super-DHB (5 mg/mL in 50% ACN and 1 mM NaOH). The spots were dried by air at room temperature. For each spot, a mass spectrum was recorded in the range from *m/z* 1,000 to 5,000, combining 10,000 shots in a random walk pattern at 1,000 Hz and 200 shots per raster spot. Prior to the analysis of the samples, the instrument was calibrated using a peptide calibration standard (Bruker Daltonics). Tandem mass spectrometry (MALDI-TOF/TOF-MS/MS) was performed for the most abundant glycans using laser-induced dissociation, and compositions as well as structural features of *N*-glycans were assessed on the basis of the observed fragment ions.

### Data Processing

For automated relative quantification of the released glycans, the MALDI-TOF-MS files were converted to text files and analyzed using MassyTools (version 0.1.8.1.) (43). Spectra were internally calibrated using glycan peaks of known composition with a S/N above nine, covering the *m/z* range of the glycans. Integration was performed on selected peaks from all glycans that were observed. For this, at least 95% of the theoretical isotopic pattern was included. Several quality parameters were used to assess the actual presence of a glycan i.e., the mass accuracy (between −10 and 10 ppm), the deviation from the theoretical isotopic pattern (below 25%) and the S/N (above three) of an integrated signal. Analytes were included for relative quantification when present in at least half of the technical replicates (excluding poor quality spectra), resulting in a list of 58 glycans. Finally, only glycans with an intensity covering at least 1% of the overall glycan abundance were selected, resulting in 28 glycans that were relatively quantified (as a fraction of the total glycan signal intensity).

### Statistics

We tested 27 anti-MOG positive patients with wild-type MOG and two aglycosylated variants of MOG, N31A and N31D. Each serum was tested with each MOG variant 4–5 times. A difference between two MOG variants was considered significant if the *p*-value was <0.05 of both the Quade omnibus-test and *post-hoc* test and if the difference between the MFI ratios was >1. Calculations were performed in R version 3.2.3. The Quade test was chosen as non-parametric test for paired samples and more than two groups. This test is recommended for our sample size. The Friedman test gave almost identical results (data not shown).

## Results

### Characterization of the Glycosylation Deficient Mutants

**Figure 1:**
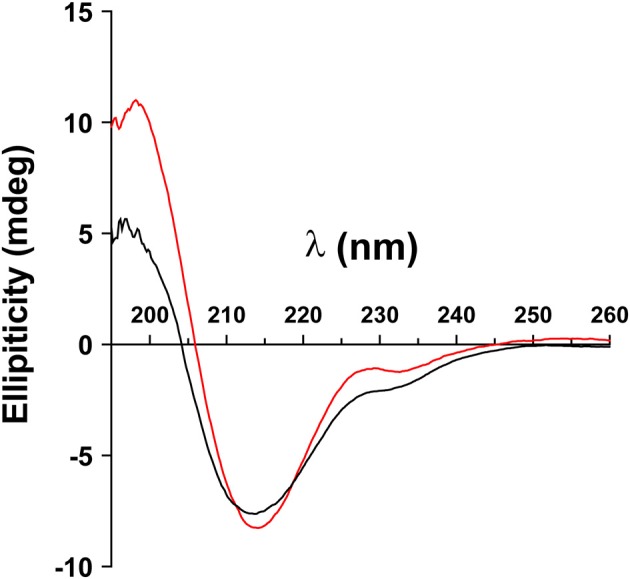
Folding of MOG does not depend on its glycosylation. The extracellular domains of wild type MOG (black line) and an aglycosylated variant (T33N; red line) were analyzed by circular dichroism. Both spectra have a similar shape representing a predominant beta sheet conformation indicated by the negative band at 213 nm. The differences around 230 and 200 nm are probably due to the presence of the avi tag in the WT, which was absent in the mutant. Protein concentration of WT and N31D was 0.1 mg/ml.

We analyzed whether the confirmation of the ECD of MOG depends on its glycosylation. To this end, we produced the ECD of wild type MOG and a mutated variant that lacks glycosylation reombinantly in HEK cells and analyzed these two proteins by circular dichroism. This showed a similar formation of beta-sheets indicating that the confirmation of MOG does not depend on its glycosylation (Figure 1).

**Figure 2:**
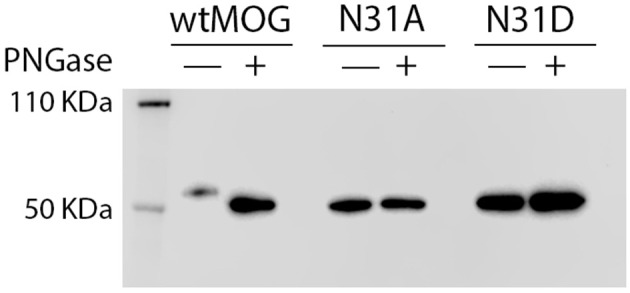
N31A and N31D mutations completely abrogate MOG glycosylation. Cell lysates of HeLa cells transiently transfected with the mutants N31A, N31D, or wild-type MOG were digested with PNGase F as indicated. Subsequently, proteins were separated by SDS gel, blotted and developed with anti-GFP-HRP antibody. The bands represent the fusion protein of MOG and EGFP.

**Figure 3:**
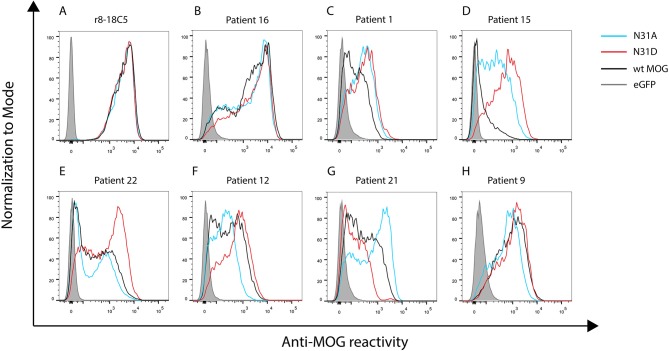
Seven patterns of anti-MOG reactivity in patients to N31A and N31D, but unaltered reactivity of r8-18C5. HeLa cells were transfected with EGFP alone (closed gray graph), wild type MOG (black line), N31A (blue line), or N31D (red line). Depicted is the reactivity of r8-18C5 and of seven patients, who represent the different pattern of anti-MOG reactivity (Table 2). One representative experiment of 4–5 replicates is shown. **(A-H)** Patterns of anti-MOG reactivity.

Lysates of cells transfected with wild type MOG or with the mutants, N31A, and N31D, each fused to EGFP were treated with PNGaseF. Cell lysates were separated by SDS-PAGE, blotted and developed with anti-GFP mAb. Under the conditions chosen for our study, wild type MOG and also both deglycosylated forms of MOG appeared only as a monomer (Figure 2). PNGaseF treatment reduced the size of MOG while the sizes of the mutated variants N31A and N31D were not changed (Figure 2). This showed that N31A and N31D are not glycosylated and that N31 is the only N-linked glycosylation site used. To see, whether the introduced mutations induced a gross alteration of MOG, both mutants were analyzed for recognition by r8-18C5 using our cell-based assay. We observed a similar expression and binding to r8-18C5 (Figure 3A).

### Heterogeneous Response to Two Glycosylation Deficient MOG Mutants

We tested 27 anti-MOG positive patients (Table 1) with wild-type MOG and two non-glycosylated variants of MOG, N31A, and N31D. About 60% of these patients (16/27) reacted to at least one of the two mutants differently than to the wild type MOG. The raw data of the reactivity of each patient to each mutant are given in Table 2 and FACS data for selected patients are shown Figure 3.

**Table 2:** Heterogeneous response to two glycosylation deficient MOG mutants.

**Patient ID**	**MFI ratio MOG**	**MFI ratio N31A**	**MFI ratio N31D**	***p*-value WT vs. N31A**	***p*-value WT vs. N31D**	***p*-value N31A vs. N31D**
**WT = N31A = N31D**						
2	6.0	7.4	5.6	0.506	0.506	1.000
4	29.0	34.2	32.9	0.506	0.506	1.000
6	211.2	164.3	199.9	0.297	1.000	0.297
10	142.4	138.3	168.5	0.574	0.083	0.188
16	187.2	225.0	211.1	0.622	0.203	0.399
18	3.7	3.9	4.6	0.390	0.060	0.214
20	5.7	7.7	7.7	0.049	0.058	0.910
23	3.7	3.6	4.4	0.064	0.039	0.003
25	97.5	114.4	117.6	0.058	0.049	0.910
26	132.5	103.9	133.7	0.161	0.781	0.108
27	77.5	86.7	121.2	0.897	0.227	0.190
**WT < N31A = N31D**						
1	9.1	16.8	18.9	0.022	0.008	0.500
7	2.6	6.0	5.3	0.002	0.034	0.034
8	44.3	70.5	90.1	0.047	0.017	0.473
11	93.2	124.7	117.4	0.008	0.022	0.500
17	27.9	89.9	56.5	0.002	0.025	0.112
19	5.9	7.5	8.2	0.024	0.005	0.337
**WT < N31A < N31D**						
15	9.8	13.9	47.4	0.034	0.002	0.034
**WT = N31A < N31D**						
3	28.5	28.0	36.6	0.325	0.022	0.005
5	80.6	77.0	108.3	0.894	0.013	0.017
14	28.9	30.6	40.7	0.500	0.008	0.022
22	45.8	42.0	102.8	0.112	0.025	0.002
24	14.5	14.4	18.9	0.337	0.024	0.005
**N31A < WT < N31D**						
12	38.8	31.4	70.1	0.034	0.034	0.002
13	94.2	65.7	102.1	0.034	0.034	0.002
**WT < N31D < N31A**						
21	15.1	45.5	7.7	0.034	0.034	0.002
**WT = N31D > N31A**						
9	26.1	20.9	27.5	0.042	0.625	0.019

*Mean fluorescence intensity (MFI) ratios were calculated as described in materials and methods; values represent the arithmetic mean of 4–5 experiments. Highlighted in gray are values considered significant. Patients (#20 and #25) had a p-value <0.05, but the response to the mutants was overall considered not significant since they did not pass the Omnibus test. Also patients #7 and #23 had p-values <0.05, but also these responses were not considered significant, because their differences of the MFI ratios were <1*.

We noted seven different patterns of reactivity toward the different non-glycosylated variants of MOG (Table 2 and Figure 3). In 11/27 patients we saw no significant difference in recognition of these MOG mutants (example in Figure 3B). In 7/27 patients a higher reactivity to both non-glycosylated MOG variants was observed. A closer look at the reactivity of these seven patients showed a further diversity. Six of these seven patients responded to the two mutants similarly (Figure 3C), while another one had a higher reactivity to N31D than to N31A (#15) (Figure 3D). In five other patients we noted a higher reactivity to N31D than to wt MOG, while the reactivity to N31A was not higher than to wt (Figure 3E). Two patients (#12 and #13) showed an increased recognition of N31D, but had a reduced reactivity for the N31A (Figure 3F). An enhanced reactivity to N31A, but a reduced one to N31D was observed in one patient (#21) (Figure 3G). Patient #9 showed a reduced reactivity to N31A (Figure 3H). Together, the reactivity to N31A was higher in 8/27 and lower in 3/27 patients, while the reactivity to N31D was higher in 14/27 and lower in only 1/27 patients. Looking at individual patients, this study reveals an enormous heterogeneity of human autoantibodies to MOG with seven different patterns of recognition uncovered by two mutations of the glycosylation site.

### Glycoforms of MOG

**Figure 4:**
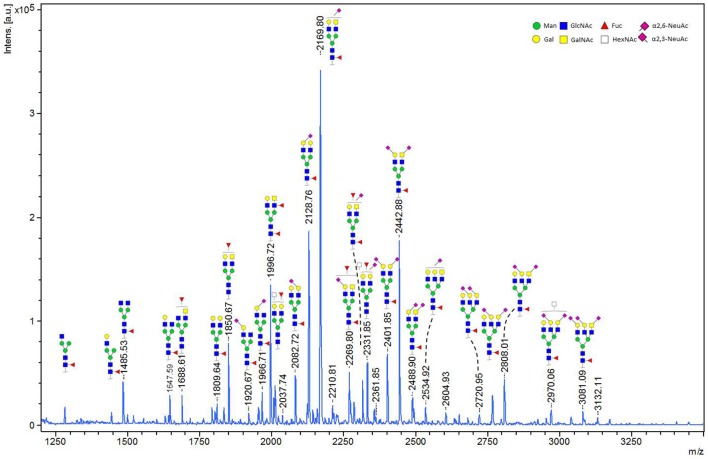
MALDI-TOF-MS spectrum of in-solution released N-glycans from recombinant MOG. Ions represent sodiated species ([M+Na]+). MALDI-TOF-MS spectrum of in-solution released *N*-glycans from recombinant MOG. Ions represent sodiated species ([M+Na]+). The compositions of the major glycan peaks were annotated based on the *m/z* values and information from tandem MS spectra (data not shown). Next to the proposed glycan structures schematically represented in the figure, additional structural isomers may be present for many of the observed glycan compositions.

**Figure 5:**
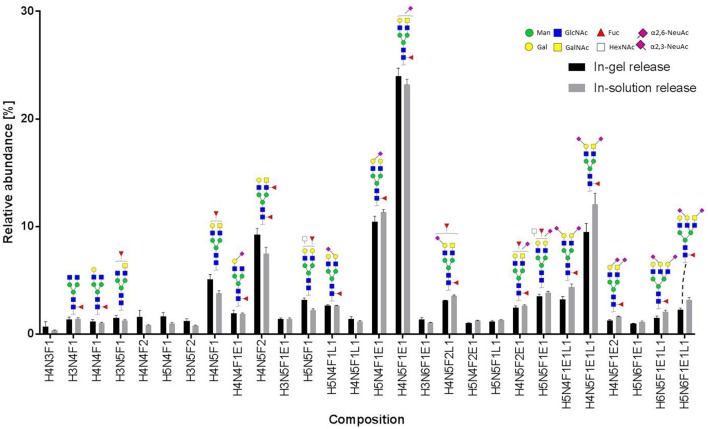
Relative abundance of recombinant MOG released N-glycans. In total, three spots from an in-gel digestion and four spots from an in-solution release were analyzed. The graph shows the average relative abundances observed for 28 glycan species (normalized to the overall sum of intensities). Abbreviations used are hexose (H), N-acetylhexosamine (N), fucose (F), and N-acetylneuraminic acid with either α2,3-linkage as indicated by lactonation (L) or α2,6-linkage as indicated by esterification (E). Error bars, standard deviation.

We performed in-gel and in-solution enzymatic release of *N*-glycans from HEK derived MOG. The sialic acid stabilized *N*-glycans were analyzed with MALDI-TOF-MS. A representative MS spectrum is shown in Figure 4. To confirm our structural assignment, we subjected several *m/z* values to tandem mass spectrometry (MALDI-TOF/TOF-MS/MS, data not shown). For example, this proved informative with regard to antenna composition and fucosylation. Most spectra showed the presence of a core fucose, where the precursor showed a loss of the reducing end *N*-acetylglucosamine together with the fucose (367.2 Da). Antenna fucosylation was observed on both LacDiNAc and LacNAc antennae, resulting in the loss of 552.1 and 511.1 Da, respectively. Additionally, the presence of LacDiNAc was confirmed by the specific fragment at *m/z* 429.3. The MS/MS spectrum of the most abundant peak at *m/z* 2169.8 showed signal losses of 725.1 Da (LacDiNAc antenna carrying an α2,6-linked sialic acid) and 684.3 Da (LacNAc antenna carrying an α2,6-linked sialic acid). This indicated a mixture of two isomers, with the sialic acid either on the LacDiNAc or LacNAc antenna. In general, the presence of bisection of glycans could not be excluded (indicated with the white squares in Figures 4, 5).

In total 28 glycans were selected for relative quantification (Figure 5). Most N-glycans were diantennary, with mainly LacNAc antennae as well as significant amounts of LacDiNAc antennae. The major glycans were sialylated species with predominantly 2,6-linked sialic acids. Most glycans showed core fucosylation, with some indications of additional antennary fucosylation. The glycan profiles obtained from in-solution and in-gel glycan release were highly consistent and showed only minor differences.

## Discussion

This study revealed that the glycosylation site of MOG influences its recognition by autoantibodies in about 60% of patients. We used two different glycosylation-deficient variants of MOG (N31D and N31A) and found seven different patterns of reactivity. While previous studies had noted that the N31D mutant was stronger recognized by some patients (1, 23, 29, 34), we now address the issue whether this is due to the introduced negative charge or due to the loss of the sugar part. Our study shows that both the negatively charged aspartate and the missing sugar can affect antigen recognition, in a different way in different patients.

Specifically, we noted that five patients showed a higher reactivity to N31D, while the reactivity to N31A was the same as to the wild type. In two other patients we observed a higher reactivity to N31D, but a lower one to N31A. We conclude that in these patients the introduced negative charge is responsible for the enhanced binding to MOG rather than the absence of the glycan.

In seven other patients, we observed a stronger reactivity to both N31D and N31A. Six of these patients showed a similarly enhanced reactivity to both mutants, while one recognized N31D stronger than N31A. One further patient showed a higher reactivity to N31A, but even a lower one to N31D. We conclude that in these 7/27 patients with an enhanced reactivity to N31A the glycan on MOG provides a hindrance for antibody binding, reminding of the impact of the glycan shield of HIV and SIV (26, 27). We then determined the glycan structure of MOG produced in HEK cells by mass spectrometry and found that the most abundant glycoforms are diantennary, contain a core fucose, an antennary fucose and are decorated with α2,6 linked Neu5Ac. Our findings indicate that this glycan structure can provide a steric hindrance for antibody binding; this might have implications for further improvement of cell-based assays to detect MOG antibodies suggesting that the use of a neutral glycosylation-deficient MOG mutant (like N31A) would enhance the sensitivity to detect autoantibodies to MOG. In none of the patients the reactivity to MOG depended on the glycan structure, clearly different than it was described for recognition of contactin (25).

Further, the MOG-reactivity of these patients is heterogeneous concerning the impact of the negatively charged N31D. One out of 27 patients showed a slightly lower reactivity to N31A, but still a clear reactivity to this glycosylation-deficient mutant. Thus, in this patient, the glycan on MOG might slightly enhance its binding to the protein-backbone. Our observation that the prototype anti-MOG r8-18C5 was not affected by any of the glycosylation deficient mutants is in accordance with the previous reports (22, 23). Our identification of seven different patterns of reactivity just using different mutations of the glycosylation site extends the knowledge about heterogeneity of MOG-epitopes recognized by patient antibodies.

While our experiments revealed the importance of the glycosylation site for antibody recognition, details how the glycan structure impacts antibody recognition remain to be identified. This could be done by altering the glycan composition by inducing or suppressing key glycosyltransferases. This may tell whether tetra-antennary versus bi-antennary glycans or differences e.g., in sialic acid linkage or branch fucosylation have an impact on antibody recognition.

Those patients who show a different reactivity to N31D and/or N31A might directly recognize the BC-loop of MOG, where the *N*-linked glycosylation site is located (23), but we cannot exclude that mutations of N31 of MOG have far-reaching effects on other parts of MOG with an impact on antibody binding at a remote side. An example for an alteration of protein-protein bindings remote from the mutation site, is the recent observation that a variant of alpha-1 antitrypsin at one side (aa213) affects the interaction of a remote part of the molecule (aa143-153) with the enzyme it inhibits, neutrophil elastase (44).

While this study elaborated the importance of the glycosylation of MOG for antibody binding, also the glycosylation of antibodies has major impact on their biological activity, both on the effector functions and on antigen-recognition. Glycosylation of the Fc-part of antibodies regulates complement activation and FcR binding (45, 46) and may serve as biomarker in autoimmunity (47). In multiple sclerosis, IgG-Fc glycosylation is altered in the CSF and indicates a pro-inflammatory pattern (48). Glycosylation of the Fab part of Ig may enhance or reduce antigen binding (49).

Glycans regulate protein-protein interactions. In an intriguing paper, glycosylation of MOG on myelin has been linked to binding to DC-SIGN and a role for myelin glycosylation in immune homeostasis of the healthy CNS was shown (50). That study further showed that removal of fucose from myelin reduced the DC-SIGN-dependent homeostatic control of myelin (50). The glycosylation in a cultured cell line may not reflect the native glycosylation of MOG in myelin. The identification of the glycoforms of MOG in myelin may help to identify binding partners of MOG. Whether MOG also interacts with sialic acid binding proteins such as Siglecs (sialic acid-binding immunoglobulin like lectins) (51) remains to be analyzed.

Together, this study shows the importance of the glycosylation site of MOG for binding of autoantibodies. Our finding that the glycan provides a hindrance for antibody binding in a proportion of patients has implications for development of assays to enhance the sensitivity to detect antibodies to MOG. Our observation of seven different patterns of MOG-binding to glycosylation-deficient variants provides further insight into details of antigen-recognition and extends the known heterogeneity of human autoantibodies against MOG.

## Data Availability

All datasets generated for this study are included in the Manuscript.

## Ethics Statement

Informed consent was obtained from each donor according to the Declaration of Helsinki and the ethical committee of the medical faculty of the LMU approveds this study.

## Author Contributions

IMF, CM, PH, AH, SW, AV, and FK performed experiments and analyzed data. DJ, RH, MW, TK, and EM designed the study and analyzed data. AK, LG, and TK provided clinical data and analyzed data. MK performed statistical analysis. All authors contributed to drafting of the manuscript.

### Conflict of Interest Statement

The authors declare that the research was conducted in the absence of any commercial or financial relationships that could be construed as a potential conflict of interest.
